# Supplementary material for: Global Impairment of Immediate-Early Genes Expression in Rett Syndrome Models and Patients Linked to Myelination Defects
Source: Int J Mol Sci. 2023 Jan 11;24(2):1453. doi: 10.3390/ijms24021453 (PMC9864472; doi:10.3390/ijms24021453)
Supplement: Supplementary file 1 [file ijms-24-01453-s001.zip › ijms-1901685-SI.pdf]

Supplementary Materials:

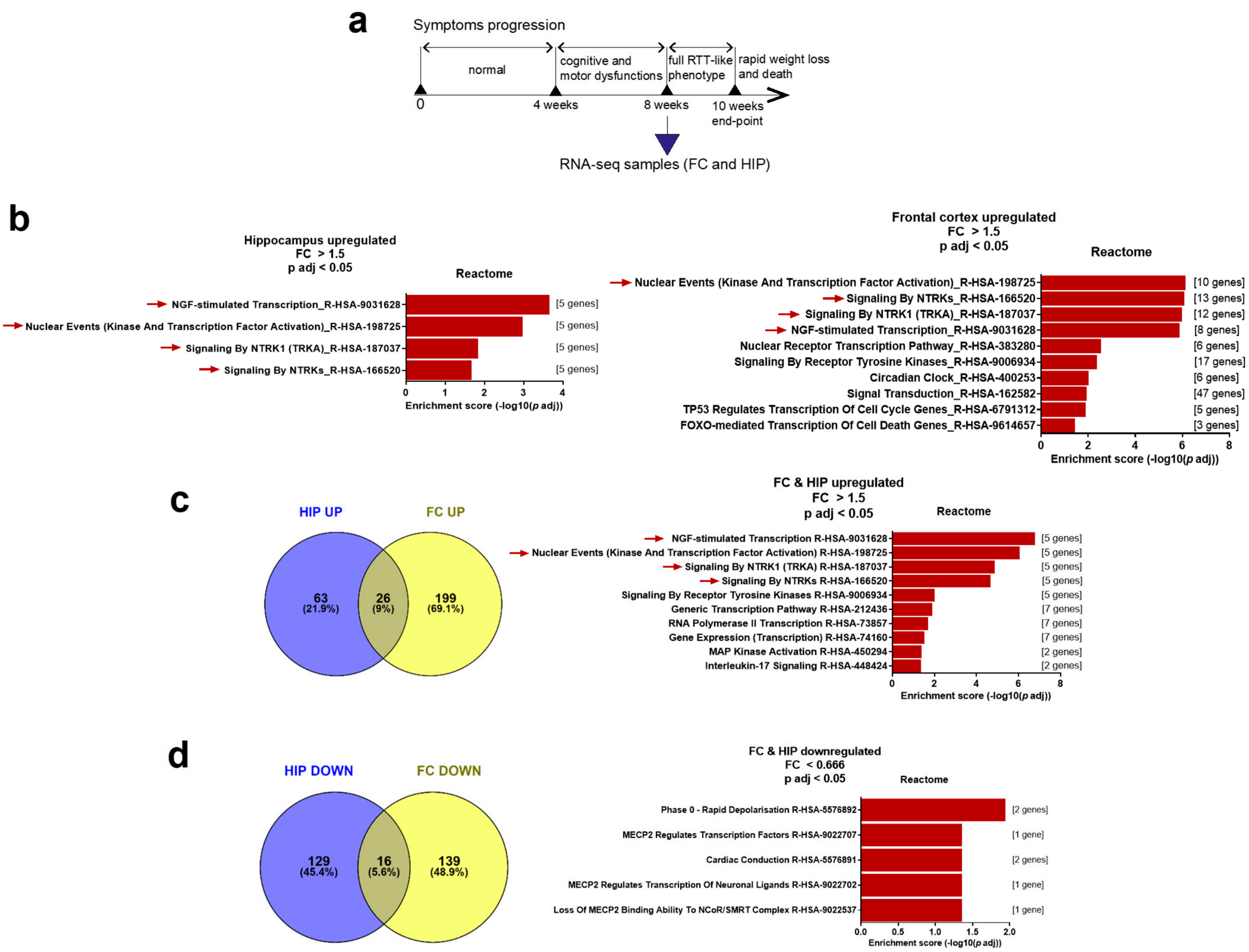

Figure S1. Differentially expressed genes (DEG) analysis in the *Mecp2*-KO(*Mecp2*$^{y/-}$) mouse model by RNA-seq. (**a**) Timeline of symptoms progression in the Rett mice. Samples for RNA-seq analysis were taken from the hippocampus and frontal cortex at 8 weeks, when the RTT-like phenotype is fully manifested. (**b**) Enrichment analysis (Reactome) of significantly upregulated genes in the hippocampus or frontal cortex (FC>1.5, adj p adj<0.05) of 8-week mice. All five significantly enriched categories in the hippocampus are represented by the following genes: *Egr1, Egr2, Arc, Fosb, Fos.* (**c**) Venn's diagram analysis shows common upregulated genes in the hippocampus and frontal cortex, together with their corresponding enriched categories. The 26 common genes are: *Mybpc1, Runx2, Etnppl, Btg2, Ciart, Fam46a, Egr1, Cdk6, Pdlim1, Zfhx3, Dusp1, Cdr1, Gm42730, Fosb, Bmper, Dusp5, Gm28036, Irak1, Nr4a1, Npas4, Egr2, Gm2260, Junb, Fos, Xlr3b, Arc.* (**d**) As in (c), but with downregulated genes. The 16 genes are: *Mecp2, Capn11, Epn3, AW551984, Grp, Gm6206, Scn7a, Fgf11, Iigp1, Igfbp2, Kirrel2, Htr1a, Gbp3, Pdyn, Cplx3, Tmem91.*

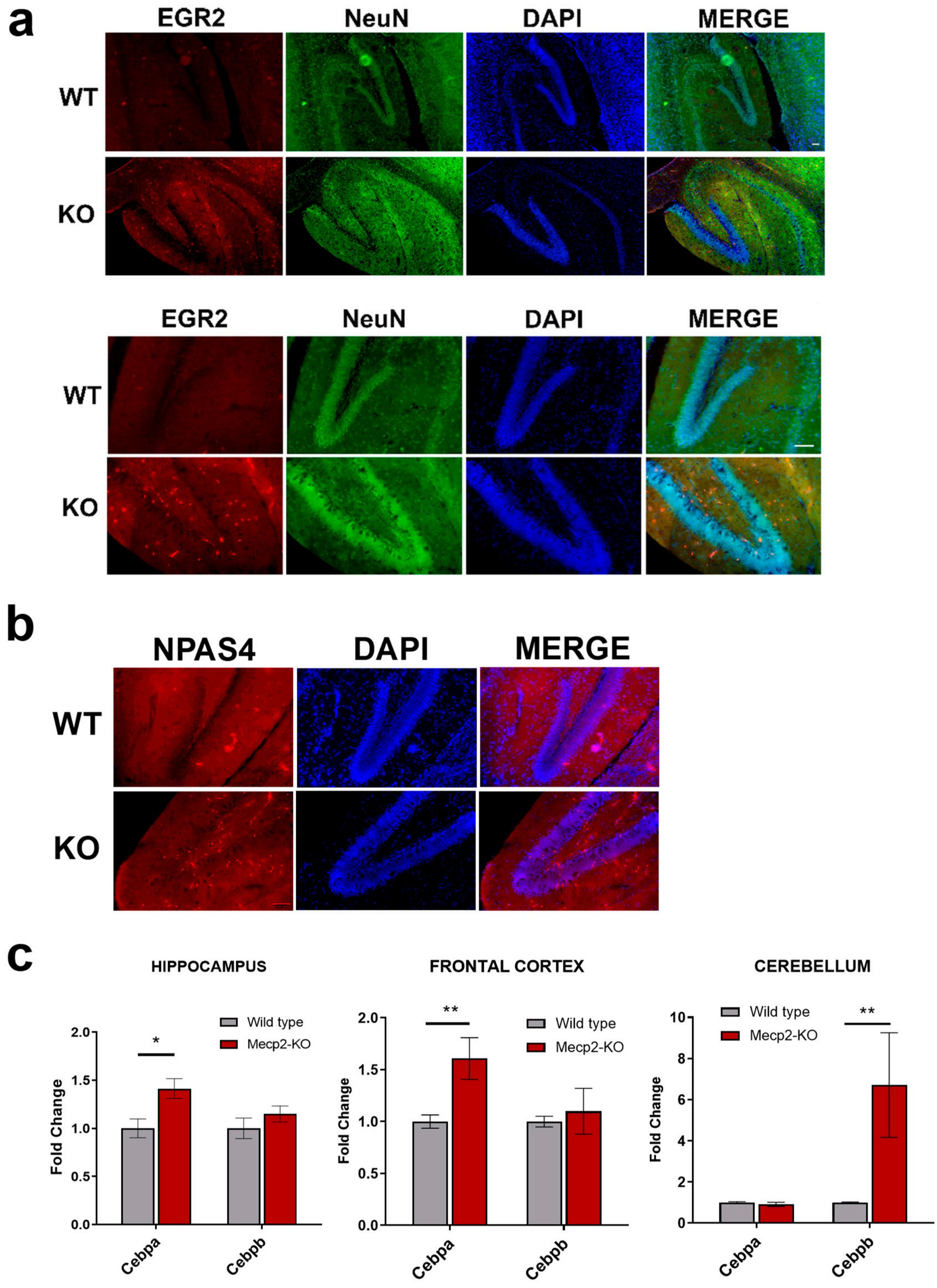

Figure S2. (a) Representative immunostaining of EGR2 protein (red) and NeuN as a neuronal marker (green) in the hippocampal regions of WT or *Mecp2*-KO 8-week mice, counterstained with DAPI (blue). Images were taken with 10x (above) or 20x (below) objectives. Scale bars represent 200 µm. (**b**) Representative immunostaining of NPAS4 protein (red) in the hippocampal regions of WT or *Mecp2*-KO 8-week mice, counterstained with DAPI (blue). Scale bar represents 160 µm All images were processed with ImageJ Fiji version 1.50g. (**c**) Analysis of *Cebpa* and *Cebpb* gene expression in the indicated regions of WT or *Mecp2*-KO 8-week mice by RT-qPCR (n = 3 animals per condition, graphs represent means ± SEM, * $p < 0.05$ in Student's t-tests).

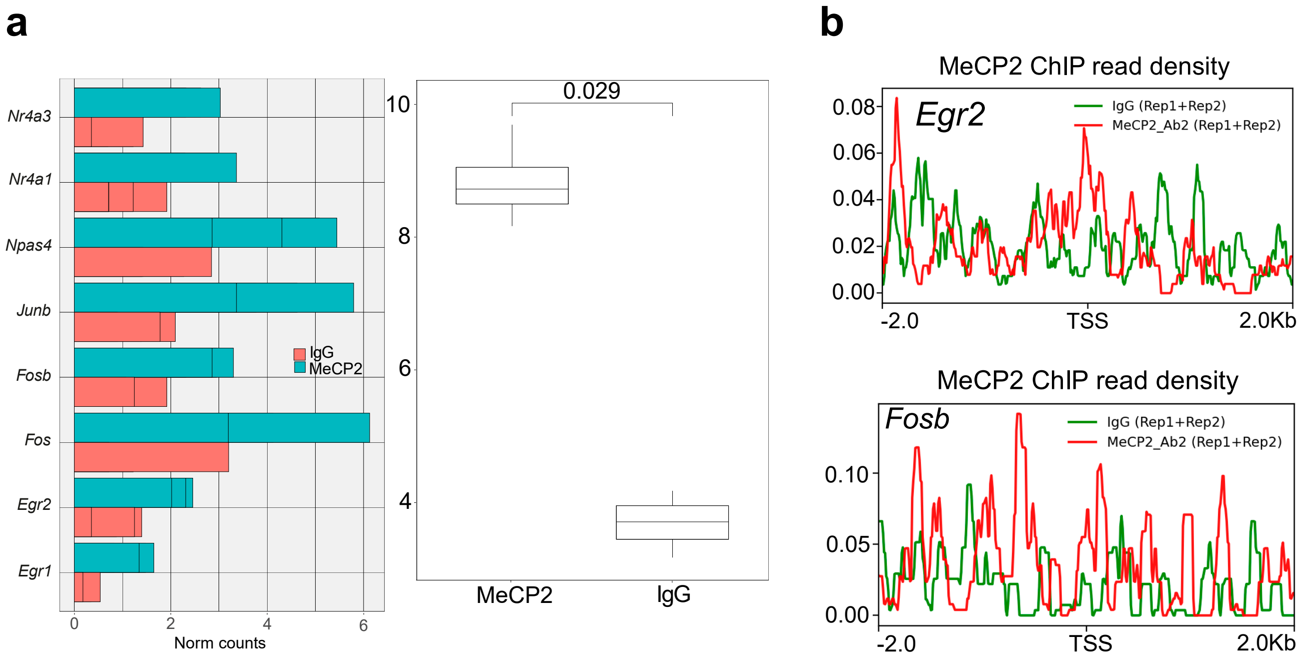

Figure S3. MeCP2 is enriched on IEGs promoters in public datasets. (**a**) Distribution of normalized ChIP-seq counts on the indicated IEGs promoters. Counts were normalized to counts per million (cpm) and a Wicoxon-ranked test was conducted for comparison between the control and the MeCP2 group, showing a significant difference. (**b**) As examples, the MeCP2 ChIP-seq read distribution profile encompassing the transcription start site (TSS) is shown for *Egr2* and *Fosb* genes.

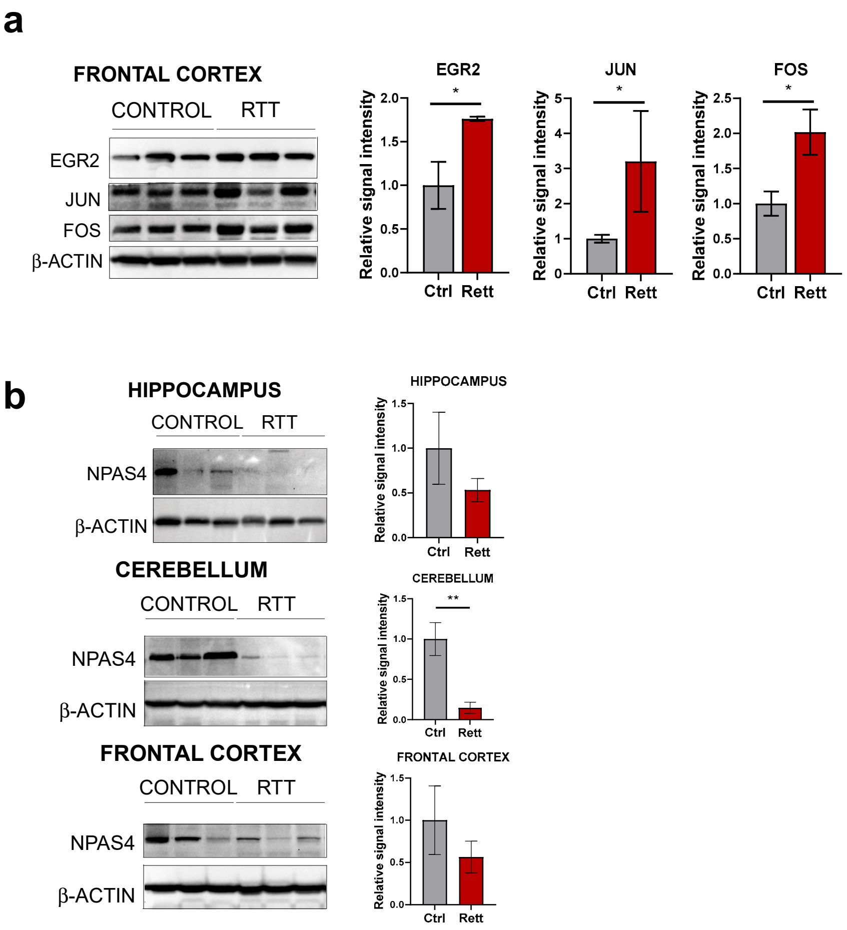

Figure S4. (**a**) Western Blot analysis of EGR2, JUN and FOS protein levels in the frontal cortex of post-mortem RTT patients' samples. Actin was used as loading control. Graphs on the right indicate the quantitation of band intensity (mean values ± SEM of n = 3 subjects per condition). Student's *t*-tests were used (* $p < 0.05$). (**b**) Western Blot analysis of NPAS4 protein levels in the indicated brain regions of post-mortem RTT patients' samples. Graphs on the right indicate the quantitation of band intensity (mean values ± SEM of n = 3 subjects per condition). Student's *t*-tests were used.

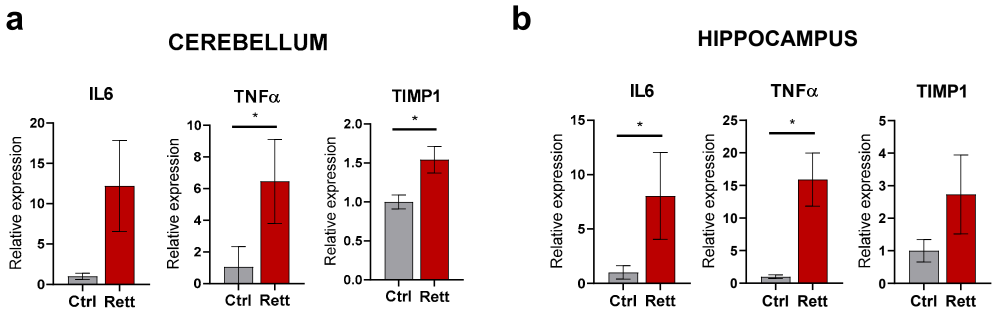

Figure S5. Expression levels of pro-inflammatory cytokines in RTT patients. (**a, b**) *IL6*, *TNFα*, and *TIMP1*mRNA levels were measured by RT-qPCR in the cerebellum (**a**) or hippocampus (**b**) of post-mortem RTT patients or healthy control samples (n = 3 subjects per condition, graphs represent means ± SEM, * $p < 0.05$ in Student's t-tests).
